# Bisphosphonate Use and Cardiovascular Outcomes According to Kidney Function Status in Post-Menopausal Women: An Emulated Target Trial from the Multi-Ethnic Study of Atherosclerosis

**DOI:** 10.3390/diagnostics15131727

**Published:** 2025-07-07

**Authors:** Elena Ghotbi, Nikhil Subhas, Michael P. Bancks, Sammy Elmariah, Jonathan L. Halperin, David A. Bluemke, Bryan R Kestenbaum, R. Graham Barr, Wendy S. Post, Matthew Budoff, João A. C. Lima, Shadpour Demehri

**Affiliations:** 1 Department of Radiology and Radiologic Sciences, Johns Hopkins University School of Medicine, Baltimore, MD 21287, USA; 2 Division of Public Health Sciences, Department of Epidemiology & Prevention, Wake Forest University School of Medicine, Winston-Salem, NC 27109, USA; 3 Division of Cardiology, University of California, San Francisco, CA 94143, USA; 4 The Cardiovascular Institute, Mount Sinai Medical Center, New York, NY 10029, USA; 5 Department of Radiology, University of Wisconsin-Madison School of Medicine and Public Health, Madison, WI 53706, USA; 6 Division of Nephrology, Department of Medicine, University of Washington, Seattle, WA 98195, USA; 7 Department of Medicine, Columbia University Medical Center, New York, NY 10032, USA; 8 Division of Cardiology, Department of Medicine, Johns Hopkins University School of Medicine, Baltimore, MD 21287, USA; 9 Lundquist Institute at Harbor-University of California Los Angeles School of Medicine, Torrance, CA 90502, USA

**Keywords:** bisphosphonates, coronary artery calcium (CAC) score, post-menopausal women, glomerular filtration rate (GFR)

## Abstract

**Background/Objectives:** Bisphosphonates may influence vascular calcification and atheroma formation via farnesyl pyrophosphate synthase inhibition in the mevalonate pathway regulating bone and lipid metabolism. However, the clinical impact of NCB use on cardiovascular outcomes remains uncertain, largely due to methodological heterogeneity in prior studies. We aimed to evaluate the association between nitrogen-containing bisphosphonate (NCB) therapy and coronary artery calcium (CAC) progression, as well as the incidence of cardiovascular disease (CVD) and coronary heart disease (CHD) events. **Methods:** From 6814 participants in MESA Exam 1, we excluded males (insufficient male NCB users in the MESA cohort), pre-menopausal women, baseline NCB users, and users of hormone replacement therapy, raloxifene, or calcitonin. Among 166 NCB initiators and 1571 non-users with available CAC measurements, propensity score matching was performed using the available components of FRAX, namely age, race, BMI, LDL cholesterol, alcohol, smoking, and steroid use, and baseline CAC yielded 165 NCB initiators matched to 473 non-users (1:3 ratio). Linear mixed-effects models evaluated CAC progression, and Cox models analyzed incident CVD and CHD events. **Results:** In the overall cohort, NCB use was not significantly associated with CAC progression (annual change: −0.01 log Agatston units; 95% CI: −0.05 to 0.01). However, among participants with a baseline estimated glomerular filtration rate (eGFR) < 65 mL/min/1.73 m^2^, NCB use was associated with attenuated CAC progression compared with non-users (−0.06 log Agatston units/year; 95% CI: −0.12 to −0.007). No significant association was observed between NCB use and incident CVD events in the overall cohort (HR: 0.90; 95% CI: 0.60−1.36) or within kidney function subgroups. **Conclusions:** Incident NCB use among postmenopausal women with mild or no CAC at baseline was associated with reduced CAC progression only in women with impaired kidney function. However, this association did not correspond to a decreased risk of subsequent cardiovascular events, suggesting that the observed imaging benefit may not translate into meaningful clinical association.

## 1. Introduction

In asymptomatic individuals, the coronary artery calcium (CAC) score is an established and independent predictor of cardiovascular risk [1]. CAC scoring based on non-contrast computed tomographic (CT) imaging quantifies calcified plaque using the modified Agatston score [2]. Its utility in identifying patients at intermediate risk and guiding preventive strategies is well-established, supporting therapeutic decisions [1].

Bisphosphonates are standard therapy for the management of osteoporosis, a common comorbidity in older adults with cardiovascular disease (CVD) [3]. Newer-generation nitrogen-containing bisphosphonates (NCBs) exhibit a strong affinity for mineralized bone tissue [4]. Controversial evidence suggests that NCBs may attenuate vascular calcification and modulate atheroma formation through the inhibition of farnesyl pyrophosphate synthase, a key enzyme in the mevalonate pathway [5,6] involved in both bone and lipid metabolism [7]. The effect of NCBs on reducing vascular calcification may also be mediated by their inhibition of bone resorption, which limits the subsequent release of calcium phosphate in soft tissues from the skeletal matrix [7]. While some studies suggest that bisphosphonates may influence the progression of or even promote the regression of vascular calcification [6,8,9], others have found no significant association [10,11,12]. The effect of bisphosphonates on vascular calcification also appears to differ between medication types, such as simple bisphosphonates and nitrogen-containing bisphosphonates [4], and they also vary based on kidney function status [13].

The literature on the association between NCBs and cardiovascular outcomes is similarly inconclusive. Some studies have reported potential adverse effects [14], others indicate benefits [15], and additional investigations have found no significant association [16,17]. A 2010 cross-sectional study from the Multi-Ethnic Study of Atherosclerosis (MESA) showed that in fully adjusted models, there was no statistically significant association between NCB use and CAC [18]. Notably, this study did not include data on cardiovascular events, nor was the progression of CAC evaluated.

Given the widespread use of bisphosphonates, especially in post-menopausal women, and the conflicting evidence regarding the association between NCBs on CAC scores and vascular calcifications according to the underlying kidney function status, our study aims to investigate two key objectives, which are (1) to determine the longitudinal association between NCB use and repeated measures of CAC and (2) to estimate the association of NCB use with subsequent cardiovascular events in post-menopausal women. Comprehensive data on CAC imaging data and bisphosphonate use in successive examinations in the well-characterized and racially/ethnically diverse MESA cohort provides a robust foundation for a detailed analysis of these associations.

## 2. Methods

The MESA is a cohort study funded by the National Heart, Lung, and Blood Institute, enrolling 6814 men and women aged 45–84 from six U.S. communities (Forsyth County, NC, USA; Northern Manhattan and the Bronx, NY, USA; Baltimore, MD, USA; St. Paul, MN, USA; Chicago, IL, USA; and Los Angeles County, CA, USA). Participants were free from clinical cardiovascular disease at baseline, and those identified after enrollment to have a prior diagnosis of cardiovascular disease were excluded. During study visits, physical exams and non-invasive cardiovascular assessments were conducted by trained staff following standardized protocols. Detailed information about the MESA study design has been published previously. The MESA has been approved by the institutional review boards of all participating centers (ClinicalTrials.gov identifier: NCT00005487) [19].

The coronary artery calcium score was measured using electron-beam or multidetector CT scanners at baseline. All scans were interpreted by trained radiologists or cardiologists at the MESA CT reading center. Each participant underwent two baseline scans, and the mean Agatston score from the two scans was used in the analysis [20]. Follow-up CAC assessments were conducted on all participants at baseline and on subsamples during subsequent examinations using a 50/50 random sampling strategy for Exams 2 and 3 and targeted, non-random sampling for Exams 4 and 5.

Medication use was evaluated using a validated inventory. Participants were asked to bring all prescription and over-the-counter medications to each visit, and trained personnel recorded the medication names and dosages [18]. NCB therapy was based on the use of any oral or intravenous NCBs, including alendronate, ibandronate, risedronate, or zoledronic acid [18] during Exams 1–5.

Cardiovascular outcomes were tracked through 2019. Participants were contacted via telephone at 9- to 12-month intervals to assess health status and identify interim events, including hospital admissions, outpatient cardiovascular diagnoses, and deaths. To verify self-reported diagnoses, copies of death certificates and medical records were requested for all reported hospitalizations and outpatient cardiovascular encounters. Next-of-kin interviews were conducted in cases of out-of-hospital cardiovascular deaths. Data collection followed a standardized protocol and utilized multiple sources, including direct interviews, public records (e.g., death certificates), hospitalization records, and autopsy reports. Trained personnel abstracted relevant information from any medical records suggesting possible cardiovascular events. Each case was independently reviewed by two physicians from the MESA Events Committee to assign event classifications and determine incidence dates. Discrepancies were resolved through discussion between the reviewers; if disagreements persisted, the full committee adjudicated the final classification [19,21]. A coronary heart disease (CHD) event was defined as death due to coronary heart disease, resuscitated cardiac arrest, myocardial infarction, angina, or revascularization when accompanied by adjudicated preceding or concurrent angina. CVD events encompassed coronary heart disease death, resuscitated cardiac arrest, myocardial infarction, angina, coronary revascularization with confirmed angina, stroke (excluding transient ischemic attack), cardiovascular death, or atherosclerotic-related death [22]. The adjudication of CHD and CVD events has been described in prior MESA publications [23].

## 3. Target Trial Emulation

We emulated a target trial using observational data, following established frameworks for target trial emulation [24]. In this hypothetical trial, post-menopausal women without a history of NCB use at baseline would be randomized to initiate NCB therapy following a baseline CAC scan. Periodic follow-up CAC scans would then be conducted to monitor progression. Table 1 outlines the components of an ideal randomized clinical trial, highlighting those that have been achieved and those that have not been achieved in the design of our emulated target trial.

### 3.1. Study Population, Eligibility, and Exclusion Criteria

**Figure 1:**
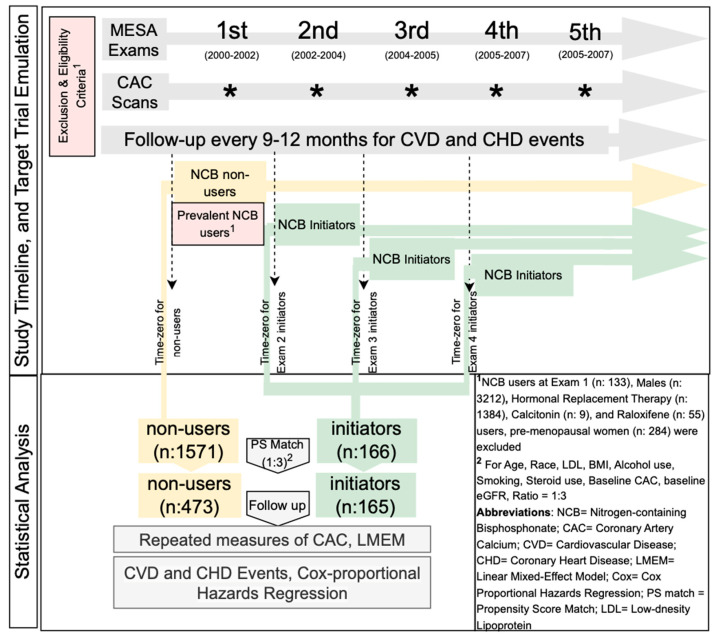
Study timeline and target trial emulation.

From the 6814 participants enrolled in the MESA at Exam 1, we excluded 133 NCB users (i.e., prevalent users) at Exam 1, 3212 males, 1384 hormone replacement therapy (HRT) users, 55 raloxifene users, 9 calcitonin users, and 284 pre-menopausal women. The final cohort comprised 166 NCB initiators and 1571 non-users with available CAC measurements during follow-up. Detailed inclusion and exclusion criteria are presented in Figure 1.

Missing data for key baseline variables of interest, including age, race, body mass index (BMI), low-density lipoprotein (LDL), alcohol use, smoking history, baseline eGFR, and steroid use, were assessed using the MCAR test (missingness < 3%, at random). For categorical variables with missing values, we created a new category labeled “missing,” allowing these cases to remain in the analysis while preserving statistical power. For numerical variables with missing values, we replaced the missing entries with the median of the respective variable. The percentage of missing values for each variable is presented in Supplementary Table S2.

### 3.2. Propensity Score Matching

We assumed two groups (initiators and non-users) were exchangeable at time zero, which was dependent on the patient-level covariates. To approximate the conditions of randomization, we employed propensity score (PS) matching [25]. We performed a multivariable logistic regression to estimate the predicted likelihood (propensity score) of initiating NCB versus non-initiation. A nearest-neighbor matching algorithm with a 1:3 ratio was used, applying a caliper width of 0.2 standard deviation of the logit of the propensity score. The propensity score models were developed using baseline variables, including components of the Fracture Risk Assessment Tool (FRAX) (age, race, BMI, alcohol use, smoking, and steroid use), LDL cholesterol, baseline CAC scores, and baseline eGFR. NCB initiation status was the treatment variable for which propensity scores were estimated. Standardized mean differences (SMDs) were checked after matching to ensure balance between groups.

### 3.3. Treatment Assignment

Treatment assignment was determined based on the initiation of NCB therapy at subsequent MESA visits, excluding individuals who were already using NCBs at Exam 1 (i.e., prevalent users). The study’s time zero for NCB initiators was defined individually for each participant as the date of the MESA visit at which NCB therapy was first initiated. For NCB initiators without a CAC measurement at the time of initiation, the closest CAC measurement from the exams prior to initiation was considered as the baseline CAC. If a participant stopped using NCB, all subsequent visits after discontinuation were excluded from the analysis.

### 3.4. Outcome

Participants were followed according to MESA rescan protocols, which included repeated CT scan assessments of CAC. Participants were monitored for incident CVD and CHD events throughout the follow-up period. The follow-up period for participants began at time zero and continued until the end of the follow-up at MESA study or death.

## 4. Statistical Analysis

### 4.1. Analysis of Association Between NCB Use and CAC

The target trial’s causal contrasts of interest involved a per protocol comparison of assignment to NCB initiator versus non-user groups. An association between NCB use and repeated measures of CAC progression employed a linear mixed-effects model (LMEM) with a random intercept for each participant. The interaction between NCB initiation, treated as a binary variable, and the time interval (in years) from baseline (time zero) to the calendar date of each CAC scan was included in the model as a main predictor. The estimated annual difference in CAC progression was reported. To assess whether the association between NCB use and CAC progression varied across levels of kidney function, stratified analyses were conducted based on the glomerular filtration rate (GFR). To determine the optimal eGFR threshold for assessing potential effect modification, we tested multiple candidate cutoffs (range: 30–95 mL/min/1.73 m^2^ in five-unit increments) using the Akaike Information Criterion (AIC) [26,27]. The threshold of 65 mL/min/1.73 m^2^ yielded the lowest AIC and was selected. Participants were subsequently stratified into two groups (GFR < 65 and GFR ≥ 65) to explore potential effect modification by kidney function. Model assumptions, including homoscedasticity, the normality of residuals, and linearity, were assessed and verified.

### 4.2. Association Between NCB Use and CVD/CHD Events

The association between NCB use and CVD or CHD events was analyzed using Cox proportional hazards regression. Models were adjusted for baseline CAC, with NCB initiation as the primary predictor and time-to-event outcomes (CVD or CHD events) as the endpoints. The proportional hazards assumption was assessed for each model using Schoenfeld residuals.

### 4.3. Sensitivity Analyses

Sensitivity analyses were conducted. First, we repeated the analyses, restricting the cohort to participants aged >65 and >75 years. Additionally, we performed the analyses on the full cohort of eligible participants, foregoing PS matching and instead adjusting the models for propensity scores as covariates, calculated based on the same baseline variables used for PS matching. Moreover, we analyzed the LMEM using the total phantom-adjusted calcium volume as the outcome instead of the Agatston score. Finally, we reanalyzed the data without excluding prevalent users at Exam 1.

## 5. Results

After propensity score matching, 165 NCB initiators were matched with 473 non-users. The baseline characteristics of the propensity score-matched groups are presented in Table 2, demonstrating excellent balance between the groups, as indicated by all SMDs being <0.1 (Supplementary Figure S1). Additionally, the baseline characteristics before matching are provided in Supplementary Table S1. The median age at baseline was 68 years (IQR: 63–74) for NCB initiators and 68 years (IQR: 60–75) for non-users. The two groups were comparable in terms of racial and ethnic composition, with BMI, LDL, HDL, diabetes status, hypertension status, use of antihypertensive medications, alcohol consumption, smoking status, and steroid use being assessed. The median baseline phantom-adjusted CAC Agatston score was three (IQR: 0–73) for NCB initiators and 0 (IQR: 0–62) for non-users. The participants in both groups had a similar number of follow-up CAC scans, with an average of 2.4 scans per group.

Compared with non-users, NCB initiation was not associated with CAC progression (estimated difference per year: −0.01; 95% CI, −0.05 to 0.01), as shown in Table 3. In subgroup analyses, NCB use was associated with a very small reduction in CAC progression among participants with eGFR < 65 (estimated difference per year: −0.06; 95% CI, −0.12 to −0.007). No association was observed for participants with eGFR ≥ 65 (estimated difference per year: 0.016; 95% CI, −0.02 to 0.05). Among participants with eGFR < 65, the median CAC Agatston score change during follow-up was 11.5/year (IQR: 1.0–55.0) for non-users and 8/year (IQR: 0–40.5) for NCB initiators. For participants with eGFR ≥ 65, the median CAC Agatston score change was 2.5/year (IQR: 0–15) for non-users and 3/year (IQR: 0–12.5) for NCB initiators.

**Figure 2:**
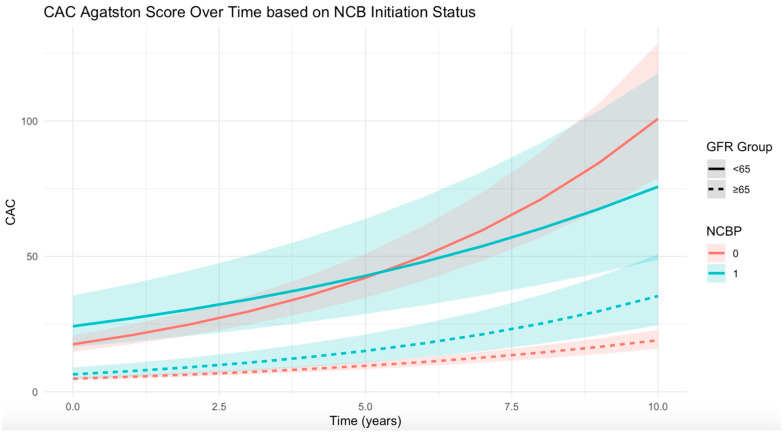
Predicted coronary artery calcification (CAC) values over a 10-year period for propensity score (PS)-matched nitrogen-containing bisphosphonate (NCB) initiators and non-users based on the estimated model coefficients. Predictions were generated on the log-transformed scale and subsequently converted to the original scale for interpretability. Abbreviations: NCB, nitrogen-containing bisphosphonate; CAC, coronary artery calcification; GFR, glomerular filtration rate; PS, propensity score.

A total of 121 CVD events (13.5 per 1000 person-years) and 67 CHD events (7.3 per 1000 person-years) occurred during follow-up. In multivariable-adjusted Cox proportional hazards models, NCB initiation was not associated with a significant difference in the risk of CVD (hazard ratio [HR], 0.90; 95% CI, 0.60–1.36) or CHD (HR, 0.92; 95% CI, 0.53–1.59; Table 4) compared with non-use. Subgroup analyses stratified by kidney function showed no significant associations between NCB use and either CVD or CHD events among participants with eGFR < 65 mL/min/1.73 m^2^ (CVD: HR, 0.91; 95% CI, 0.30–2.68; CHD: HR, 1.06; 95% CI, 0.29–3.78) or those with eGFR ≥ 65 mL/min/1.73 m^2^ (CVD: HR, 0.94; 95% CI, 0.61–1.48; CHD: HR, 0.94; 95% CI, 0.51–1.73; Table 4). Figure 2 illustrates the predicted CAC values over a 10-year period for both NCB initiators and non-users based on the estimated model coefficients. The predictions were initially made on the log-transformed scale and were subsequently converted back to the original scale for better interpretation.

The results of the sensitivity analyses are presented in Table 5. Overall, the findings remained consistent and there was no effect modification when the analyses were restricted to participants aged >65 and >75. Additionally, when models were adjusted for PSs instead of applying PS matching and when calcium volume was treated as an outcome, NCB use again showed a negative association with CAC changes in participants with eGFR < 65. However, when prevalent users were included, no association was observed between NCB use and changes in CAC across all eligible participants and eGFR strata.

## 6. Discussion

In this comprehensive study, NCB initiation was not associated with slower CAC progression in middle and older age post-menopausal women from the MESA cohort. In addition, no difference in cardiovascular events with vs. without bisphosphonate use was identified. However, renal disease has previously been thought to be a confounder in such analyses. In a stratified analysis among participants with eGFR < 65, NCB use was associated with slower CAC progression. However, the magnitude of this association was small and did not translate into a lower risk of downstream cardiovascular events, indicating that the clinical impact of NCB use in this context may be limited.

Debate is ongoing regarding the impact of bisphosphonates on CAC progression [12]. While some studies suggested no significant association [10,11,12], others presented evidence that bisphosphonates may alter the progression of CAC or contribute to its regression [6,8,9]. These inconsistencies may be attributed in part to differences in study designs and populations. An observational study of 35 patients with renal disease found that 90 days of etidronate treatment reduced CAC progression compared with the period before treatment initiation in the same population [6]. An observational study of 56 alendronate-treated patients with osteoporosis, matched to 56 control subjects, found no significant difference in CAC progression rates between the alendronate-treated group and either the matched or reference control cohorts. However, the study did not provide information on kidney function status, which may influence CAC progression and treatment effects [10]. A cross-sectional study on baseline data in MESA participants found that NCB use was associated with less calcification of the mitral anulus, thoracic aorta, and aortic valve ring among women over age 65 but higher cardiovascular calcifications in those younger than 65 [18]. In fully adjusted models, however, the association between NCB use and CAC lost statistical significance in both age groups. Building on those findings, our propensity score-matched analysis revealed no significant association between NCB use and changes in CAC across repeated measurements over time in MESA participants. These findings are consistent with those of a 2024 systematic review of four observational studies and one randomized clinical trial, including 377 patients (223 bisphosphonate users and 154 non-users) with a mean age of 66 years and follow-up period between 6 and 54 months [12]. This systematic review concluded that evidence remains insufficient to support a correlation between bisphosphonate use and CAC progression [12].

In addition to CAC progression, the impact of bisphosphonates on cardiovascular outcomes remains a topic of ongoing debate. A retrospective study of 3234 women with chronic kidney disease found that bisphosphonate treatment was associated with a lower risk of mortality but did not demonstrate a significant effect on cardiovascular events [28]. Similarly, a 2016 meta-analysis of 61 trials reported that bisphosphonates reduced arterial wall calcification but had no effect on the incidence of cardiovascular events [29]. While the meta-analysis suggested that bisphosphonates might reduce cardiovascular mortality and all-cause mortality, conclusions specific to kidney function could not be drawn. A more recent meta-analysis encompassing 69 trials found that bisphosphonate therapy did not affect all-cause mortality, adverse events, or cardiovascular events [17], but like previous studies, it lacked specific information regarding participants’ kidney function status. Our analysis observed no association between NCB initiation and cardiovascular outcomes regardless of kidney function status.

While our analysis included all participants with reported use of alendronate, ibandronate, risedronate, or zoledronic acid, over 90% of NCB users in the cohort were alendronate users. This precluded a stratified analysis by specific NCB agent or dosing regimen. Although our repeated-measures modeling framework captures CAC progression over time and accounts in part for the duration of use, the role of individual NCB types and doses on CAC progression and cardiovascular outcomes remain an important area for research.

## 7. Strengths and Limitations

This study has several strengths. First, we excluded participants with uncertain NCB initiation times (i.e., prevalent NCB users during MESA Exam 1), thereby minimizing the risk of immortal time bias, a common concern in observational studies. Second, we employed propensity score matching to emulate randomization, enhancing the comparability between treatment groups. Additionally, the use of LMEM allowed for the analysis of repeated outcome measurements, providing a more comprehensive understanding of longitudinal changes. There are also several limitations. The observational design inherently carries a greater risk of residual unmeasured confounding compared to a randomized controlled trial. Moreover, our comparison group consisted of bisphosphonate non-users rather than an active comparator group (e.g., denosumab or selective estrogen receptor modulator). While we performed baseline matching using available FRAX components and included only postmenopausal women, an ideal control group would have consisted of individuals with a medical indication for bisphosphonates but not receiving NCB. Furthermore, the follow-up duration in our study was shorter than that of typical long-term bisphosphonate treatment courses. In addition, bone mineral density data were not assessed in this analysis, precluding an evaluation of osteoporosis severity or a formal determination of treatment indication. Moreover, unlike in trials, we relied on medication inventory forms to determine bisphosphonate use, and the use of observational data limits our ability to control for all aspects of treatment adherence and follow-up. Additionally, there are limitations inherent to the MESA cohort. Participants were free from cardiovascular disease at baseline, which limits generalizability to populations with pre-existing conditions. Despite being a large multiethnic cohort with extensive follow-up and repeated CAC measurements, the MESA is not fully representative of the general population. Additionally, while MESA participants were followed comprehensively, the follow-up period was relatively short compared to the extended treatment durations among NCB users in real-world settings. Because the eGFR cutoff was selected based on AIC minimization across multiple candidate thresholds, the resulting stratification may be subject to overfitting and increased type I error. As such, findings from this subgroup analysis should be interpreted cautiously and considered exploratory until validated in external cohorts. Finally, although we observed a statistically significant reduction in CAC progression among NCB users with impaired kidney function, the magnitude of this change is modest and may fall within the range of interscan variability. The clinical significance of such small changes in CAC is uncertain to reflect meaningful reductions in cardiovascular risk.

## Figures and Tables

**Table 1:** Design elements of an ideal randomized trial vs. an emulated target trial. Initiation of NCB vs. non-users throughout the study timeline.

Protocol Components	Ideal Randomized Trial	Emulated Target Trial
Eligibility criteria	Post-menopausal women	Post-menopausal women
Exclusion criteria	Previous NCB use Calcitonin use Raloxifene use Hormonal replacement therapy	Prevalent NCB use Calcitonin use Raloxifene use Hormonal replacement therapy
Treatment strategy	Initiation of NCB ^1^ vs. placebo	Initiation of NCB vs. non-users throughout the study timeline
Assignment	Randomized and blinded	Emulated by propensity score matching (1:3)
Follow-up	Start from NCB initiation until predefined timepoints, e.g., 5 years and 10 years post-initiation with follow-up cardiac CT scans	Time zero (i.e., baseline) with NCB initiation (Exams 2, 3, 4), available follow-up cardiac CT scans within MESA
Outcome	Repeated CAC measurements from baseline (time zero) over a specified follow-up period, CVD, CHD	Repeated CAC measurement from initiation of NCB to next available cardiac CT scans
Causal contrast of interest	Per protocol effect	Observational analog to per protocol effect
Data analysis plan	Linear mixed-effects model	Linear mixed-effects model

^1^ We were not able to account for the route of bisphosphonate therapy (oral, intravenous). Abbreviations: MESA = Multi-Ethnic Study of Atherosclerosis; CAC = coronary artery calcium; NCB = nitrogen-containing bisphosphonate; CVD = cardiovascular disease; CHD = coronary heart disease.

**Table 2:** Baseline characteristics of propensity-score matched NCB initiator and non-user females.

Characteristic	Non-User, *N* = 473 ^1^	NCB ^2^ Initiator, *N* = 165 ^1^	SMD ^3^
Age (years)	68 (60, 75)	68 (63, 74)	0.03
Race/ethnicity			
White	160 (34%)	51 (31%)	0.01
Chinese	113 (24%)	44 (27%)	0.006
Black	91 (19%)	34 (21%)	0.01
Hispanic/Latino	109 (23%)	36 (22%)	0.04
BMI (kg/m^2^)	26.2 (23.5, 29.5)	25.9 (23.1, 29.4)	0.02
LDL (mg/dL)	117 (99, 136)	114 (92, 139)	0.07
HDL (mg/dL)	53 (45, 63)	52 (46, 64)	
Exam 1 diabetes mellitus by 2003 ADA fasting criteria algorithm			
Normal	348 (74%)	132 (80%)	
IFG	62 (13%)	20 (12%)	
Untreated diabetes	19 (3.8%)	1 (0.6%)	
Treated diabetes	44 (9.1%)	12 (7.3%)	
Hypertension medication			
Yes	174 (37%)	63 (38%)	
Hypertension status			
Yes	250 (53%)	80 (48%)	
Baseline CAC Agatston score phantom-adjusted	0 (0, 62)	3 (0, 73)	0.04
Seated SBP (mmHg)	133 (114, 152)	128 (114, 142)	
Consumed alcoholic beverages			
Yes	282 (60%)	101 (61%)	0.02
Smoking status			
Never	322 (69%)	116 (70%)	0.02
Former	106 (23%)	40 (24%)	0.08
Current	41 (8.7%)	9 (5.5%)	0.02
Steroid use	13 (2.7%)	3 (1.8%)	0.002

^1^ Median (IQR); *n* (%). ^2^ Alendronate, ibandronate, risedronate, zoledronic acid. ^3^ Presented for variables included in PS calculation. Abbreviations: BMI = body mass index; HTN = hypertension; CAC Score = coronary artery calcium score; SMD = standardized mean difference; PS = propensity score; LDL = low-density lipoprotein; HDL = high-density lipoprotein.

**Table 3:** Linear mixed-effects model of nitrogen-containing bisphosphonate use and repeated measures of CAC.

	Initiators ^1^		Non-Users		Estimated Difference Per Year (95% CI) ^2,3^
	(*n*)	Delta CAC Agatston Score per Year (Median (IQR))	(*n*)	Delta CAC Agatston Score per Year (Median (IQR))	
All participants	165		473		−0.01 (−0.05, 0.01)
eGFR < 65	40	8 (0, 40.5)	146	11.5 (1.0, 55.0)	−0.06 (−0.12,−0.007)
eGFR ≥ 65	125	3 (0, 12.5)	327	2.5 (0, 15)	0.016 (−0.02, 0.05)

^1^ Alendronate, ibandronate, risedronate, zoledronic acid. ^2^ Main predictor: NCB initiation (binary) and time interval (years) interaction. ^3^ Outcome: Log-transformed CAC score. Abbreviations: CAC = coronary artery calcium; CI = confidence interval; eGFR = estimated glomerular filtration rate; NCB = nitrogen-containing bisphosphonate.

**Table 4:** Association of nitrogen-containing bisphosphonate use with CVD and CHD events (Cox proportional hazards regression analysis).

		CVD ^1,2,3^		CHD ^1,2,4^
	(*n* Events)	HR (95% CI)	(*n* Events)	HR (95% CI)
All participants	121	0.90 (0.60, 1.36)	67	0.92 (0.53, 1.59)
eGFR < 65	23	0.91 (0.30, 2.68)	15	1.06 (0.29, 3.78)
eGFR ≥ 65	98	0.94 (0.61, 1.48)	52	0.94 (0.51, 1.73)

^1^ All models have been adjusted for baseline CAC. ^2^ Alendronate, ibandronate, risedronate, zoledronic acid. ^3^ Coronary heart disease death, resuscitated cardiac arrest, myocardial infarction, angina, coronary revascularization with confirmed angina, stroke (excluding transient ischemic attack), cardiovascular death, or atherosclerotic-related death. ^4^ Coronary heart disease death, resuscitated cardiac arrest, myocardial infarction, angina, or revascularization with confirmed angina. Abbreviations: CVD = cardiovascular disease; CHD = coronary heart disease; CI = confidence interval; HR = hazard ratio; eGFR = estimated glomerular filtration rate; CAC = coronary artery calcification.

**Table 5:** Sensitivity analyses, linear mixed-effects model of nitrogen-containing bisphosphonate initiation/use and repeated measures of coronary artery calcium Agatson score and calcium volume.

Restricted to age > 65 at baseline, NCB initiators vs. PS-matched non-users			
	Initiators ^1^ (*n*)	Non-users (*n*)	Estimated difference per year (95% CI) ^2,3^
All	88	246	−0.03 (−0.08, 0.008)
eGFR < 65	35	102	**−0.07 (−0.13,−0.01)**
eGFR ≥ 65	53	144	−0.007 (−0.06, 0.05)
**Restricted to age > 75 at baseline, NCB initiators vs. PS-matched non-users**			
	Initiators ^1^ (*n*)	Non-users (*n*)	Estimated difference per year (95% CI) ^2,3^
All	21	61	−0.06 (−0.14, 0.01)
eGFR < 65	12	28	**−0.1 (−0.2, −0.004)**
eGFR ≥ 65	9	33	−0.01 (−0.12, 0.10)
**NCB initiators vs. non-users, adjusted for PSs ^4^**			
	Initiators ^1^ (*n*)	Non-users (*n*)	Estimated difference per year (95% CI) ^2,3^
All	166	1571	0.004 (−0.02, 0.03)
eGFR < 65	40	405	**−0.04 (−0.08, −0.003)**
eGFR ≥ 65	126	1166	0.02 (−0.01, 0.05)
**NCB initiators vs. non-users, outcome: calcium volume**			
	Initiators ^1^ (*n*)	Non-users (*n*)	Estimated difference per year (95% CI) ^2,3^
All	165	473	−0.005 (−0.04, 0.29)
eGFR < 65	40	146	**−0.06 (−0.12, −0.007)**
eGFR ≥ 65	125	327	0.015 (−0.03, 0.06)
**NCB users vs. PS-matched non-users**			
	Users ^1,5^ (*n*)	Non-users (*n*)	Estimated difference per year (95% CI) ^2,3^
All	297	748	−0.003 (−0.03, 0.02)
eGFR < 65	97	230	−0.02 (−0.06, 0.01)
eGFR ≥ 65	200	518	0.01 (−0.01, 0.04)

^1^ Alendronate, ibandronate, risedronate, zoledronic acid. ^2^ Main predictor: NCB initiation (binary) and time interval (years). ^3^ Outcome: Log-transformed CAC score. ^4^ Analyses were conducted on all eligible participants without PS matching; models were adjusted for propensity score values. ^5^ Prevalent NCB users at Exam 1 were included. Abbreviations: NCB = nitrogen-containing bisphosphonate; CAC = coronary artery calcium; CI = confidence interval; eGFR = estimated glomerular filtration rate; PS = propensity score.

## Data Availability

Data are available from the corresponding author upon request, subject to MESA data sharing guidelines.

## Supplementary Materials

The following supporting information can be downloaded at: https://www.mdpi.com/article/10.3390/diagnostics15131727/s1, Figure S1: Propensity Score Distribution for Matched Data. Density plot comparing the propensity score distributions of nitrogen-containing bisphosphonate (NCB) initiators (in orange) and NCB non-users (in blue) after matching.; Table S1: Baseline Characteristics of NCB Initiator and Non-user Females (before PS matching); Table S2: Percentages of missing values.
