# Supplementary material for: Bisphosphonate Use and Cardiovascular Outcomes According to Kidney Function Status in Post-Menopausal Women: An Emulated Target Trial from the Multi-Ethnic Study of Atherosclerosis
Source: Diagnostics (Basel). 2025 Jul 7;15(13):1727. doi: 10.3390/diagnostics15131727 (PMC12248928; doi:10.3390/diagnostics15131727)
Supplement: Supplementary file 1 [file diagnostics-15-01727-s001.zip › diagnostics-3698816-supplementary.pdf]

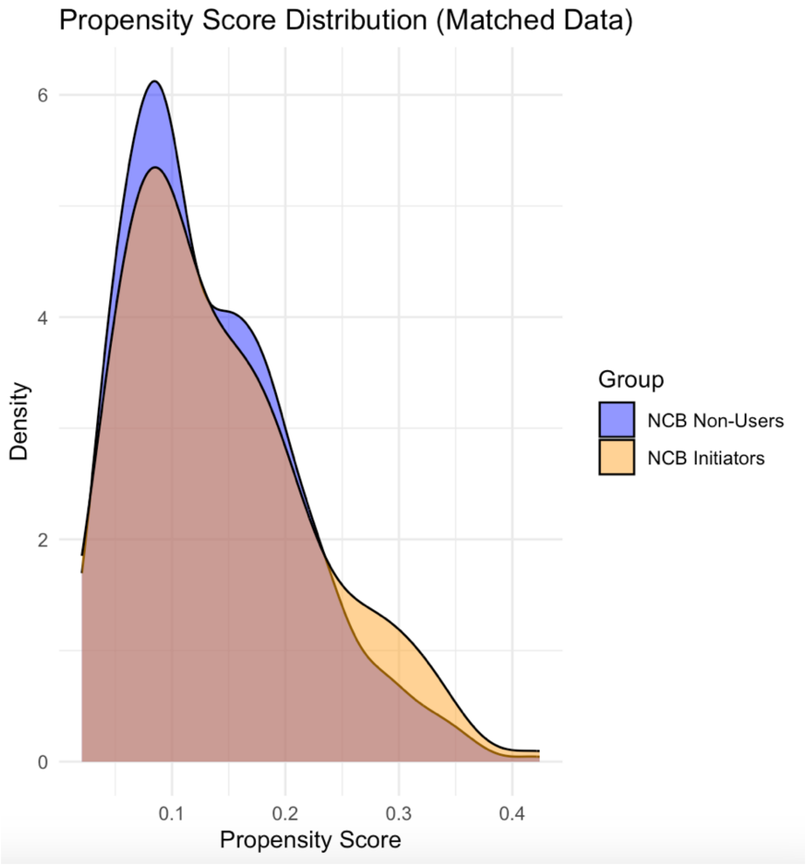

Supplementary Figure S1. Propensity score distribution for matched data. Density plot comparing propensity score distributions of nitrogen-containing bisphosphonate (NCB) initiators (in orange) and NCB non-users (in blue) after matching.

Supplementary Table S1. Baseline characteristics of NCB initiator and non-user females (before PS matching)

Characteristic	**Non-user**, N = 1,571[1]	**Initiator**, N = 166[1]	**p-value**[2]
**Age (years)**	65 (56, 72)	68 (63, 74)	<0.001
**Race/ethnicity**			<0.001
White	442 (28%)	51 (31%)	
Chinese	163 (10%)	45 (27%)	
Black	562 (36%)	34 (20%)	
Hispanic/Latino	404 (26%)	36 (22%)	
**BMI (kg/m2)**	29 (25, 33)	26 (23, 29)	<0.001
**LDL (mg/dL)**	122 (103, 144)	114 (91, 139)	0.005
**HDL (md/dL)**	52 (44, 61)	52 (46, 64)	0.079
**Exam 1 diabetes mellitus by 2003 ADA fasting criteria algorithm**			0.026
Normal	1,099 (70%)	133 (80%)	
IFG	229 (15%)	20 (12%)	
Untreated diabetes	50 (3.2%)	1 (0.6%)	
Treated diabetes	187 (12%)	12 (7.2%)	
**Hypertension medication**			0.5
Yes	640 (41%)	63 (38%)	
**Hypertension status**			0.055
Yes	823 (52%)	80 (48%)	
**CAC Agatston score phantom-adjusted**	0 (0, 51)	3 (0, 72)	0.07
**Total calcium volume phantom-adjusted**	0 (0, 53)	5 (0, 66)	0.07
**Seated SBP (mmHg)**	129 (113, 146)	128 (114, 142)	0.5

**Consumed alcoholic beverages**			0.10
No	509 (33%)	65 (39%)	
Yes	1,057 (67%)	101 (61%)	
**Smoking status**			0.011
Never	957 (61%)	117 (70%)	
Former	419 (27%)	40 (24%)	
Current	191 (12%)	9 (5.4%)	
**Steroid use**	29 (1.8%)	3 (1.8%)	0.09

[1] Median (IQR); n (%)

[2] Alendronate, Ibandronate, Risedronate, Zoledronic Acid

[3] Wilcoxon rank sum test; Pearson's chi-squared test

Abbreviations: BMI= body mass index; HTN= hypertension; CAC Score= coronary artery calcium score; PS= propensity score; LDL= low-density lipoprotein; HDL= high-density lipoprotein; NCB= nitrogen-containing bisphosphonate.

**Supplementary Table S2.** Percentages of missing values.	
Variable	Percent
Age	0%
Race	0%
BMI	0%
LDL	1.0%
HDL	0.4%
DM status	0%
HTN medication	0%
HTN status	0%
Seated SBP	0%
Consumed alcoholic beverages	0.2%
Smoking status	0.2%
Steroid use	0.1%
eGFR	0.3%
Abbreviations: BMI = body mass index; LDL = low-density lipoprotein; HDL = high-density lipoprotein; DM = diabetes mellitus; HTN = hypertension; SBP = systolic blood pressure; eGFR = estimated glomerular filtration rate.	
